# Genital and Subjective Sexual Arousal in Androphilic Women and Gynephilic Men in Response to the Copulatory Movements of Different Animal Species

**DOI:** 10.1007/s10508-024-02917-2

**Published:** 2024-08-12

**Authors:** Lucie Krejčová, Ondřej Vaníček, Martin Hůla, Kateřina Potyszová, Klára Bártová

**Affiliations:** Department of Psychology and Life Sciences, Faculty of Humanities, Charles University, Pátková 2137/5, 182 00 Prague, Czech Republic

**Keywords:** Genital response, Sexual psychophysiology, Category specificity, Sexual stimuli, Sex differences

## Abstract

Research has repeatedly shown marked differences in men’s and women’s sexual response patterns; genital response in men tends to be elicited by cues that correspond to their sexual preference (preferred gender), while women’s genital response is less sensitive to gender cues and more sensitive to the presence and intensity of other sexual cues (e.g., sexual activities). We tested whether the cue of copulatory movement in a general sexual context elicited a genital response in androphilic women but not in gynephilic men. If so, women should react to stimuli depicting not only the non-preferred gender but also other animal species differing in phylogenetic distance to humans. We studied the genital and self-reported arousal of 30 gynephilic men and 28 androphilic women to two sexual videos depicting penetrative human sexual intercourse (female-male and female-female) and nine videos depicting animal copulation. Neither women nor men showed genital or subjective sexual arousal to non-human sexual stimuli. Moreover, both sexes demonstrated a highly cue-specific pattern of arousal. Our results suggest that copulatory movement displayed in non-human species is not a sexual cue that can elicit genital or subjective sexual arousal in humans.

## Introduction

Laboratory studies of men’s and women’s sexual arousal have shown certain gender differences in the sexual cues that elicit sexual arousal. Contextual cues seem relevant to the patterns of sexual response in androphilic women (i.e., women sexually attracted to men) but not gynephilic ones (i.e., women sexually attracted to women) and in both androphilic and gynephilic men. In particular, research has repeatedly shown that both gynephilic and androphilic men’s subjective and genital arousal to erotic stimuli is category-specific. Men exhibit a higher level of genital and self-reported arousal in response to sexual stimuli that depict targets of their preferred gender (a gender-specific sexual response; Chivers, 2010), age, category, or sexual activities (a cue-specific sexual response; Blanchard et al., 2001; Timmers et al., 2018). In contrast, the pattern of genital arousal of androphilic women was found to be category-nonspecific: androphilic women react to sexual stimuli depicting targets of both their preferred and non-preferred gender even though their subjective arousal is category-specific (Chivers et al., 2007). Moreover, androphilic women show a genital response to almost any sexual cue (see Chivers, 2017 for a review). Gynephilic women tend to display similar patterns of category-specificity as men do (Chivers & Bailey, 2005; Chivers et al., 2004); however other studies have observed patterns of higher cue-specificity among gynephilic women for other responses such as pupil dilation or neural responses (see Lalumière et al., 2022 for a review).

The pattern of category-nonspecificity in androphilic women has been confirmed by various experimental tools aimed at assessing sexual response, including eye tracking (Lykins et al., 2008), pupillometry (Rieger et al., 2015), thermography (Huberman & Chivers, 2015), vaginal/clitoral photoplethysmography (Suschinsky et al., 2020), and functional magnetic resonance imaging (fMRI; Safron et al., 2020). The same effect has been observed across various stimulus modalities, including audio-visual stimuli (Chivers et al., 2007), static pictures of naked bodies (Safron et al., 2020), sexual narratives (Chivers et al., 2014; Suschinsky & Lalumière, 2011a, 2011b), and even social chemosignals obtained from potential partners with respect to gender and sexual orientation (Lübke et al., 2012).

Still, the specific features of sexual stimuli, which evoke a genital response, are yet to be thoroughly explored, especially with respect to gender differences. Physical characteristics of nude objects, such as breasts, vulva, and penis, should be among the possible cues that elicit sexual arousal. Spape et al. (2014) used vaginal photoplethysmography (VPG) to assess the genital response patterns of androphilic women exposed to images of male and female genitals. They found that depictions of erect penises produced a larger genital response than depictions of flaccid penises or exposed vulvas. Similar results were reported by Ponseti et al. (2006), who used fMRI. These findings suggest that pictures of sexually aroused genitals–even with minimal contextual cues such as sexual activity–elicit a category-specific response in androphilic women. This runs counter to the previously reported category-nonspecific arousal pattern in androphilic women.

Depiction of different types of explicit sexual activity is another possible cue that has the potential to elicit a sexual response. It has been shown that a wide variety of depictions of sexual activities between two individuals with different levels of sexual explicitness, ranging from kissing to manual or oral stimulation of genitals and penile-vaginal intercourse, increases the genital response in both men and women (Both et al., 2008; Chivers et al., 2007). On the other hand, depictions of solitary masturbation or nude physical exercise produce a weaker genital response in both genders (Chivers et al., 2007). It seems that explicit images of sexual intercourse between two individuals are sufficiently intense to produce a genital response even when the actors are of the non-preferred gender (Chivers et al., 2007).

This does not, however, explain the findings of Chivers and Bailey (2005) and Chivers et al. (2007) who exposed androphilic and gynephilic men and women to video stimuli depicting heterosexual, gay, and lesbian couples engaging in oral and penetrative sex, as well as to video stimuli of female and male bonobos engaging in penile-vaginal penetration. In these experiments, androphilic women showed a genital response to all of the presented stimuli, including mating bonobos, although they did not report being subjectively aroused. In particular, women’s genital responses to images of mating bonobos were larger than their responses to nonsexual control stimuli but significantly smaller than their responses to images of human couples. Men, on the other hand, exhibited a neither subjective nor genital response to the non-human stimulus, and the human stimuli demonstrated arousal patterns that corresponded to their stated sexual orientation. Interestingly, gynephilic women showed less category-nonspecific genital response than androphilic women did, but not to the same extent as men of either sexual orientation. A possible explanation for these results is that for men, the most important feature that elicits arousal are indicator of the actor’s gender, while for women, it is the activities and movements connected with sexual context (such as penile thrusting; Chivers & Bailey, 2005) that play a crucial role. To date, no experimental study has focused on testing these suggestions.

### The Current Study

Gender differences in the specificity of genital response may reflect the different cues displayed in the sexual stimuli. For men, the most important features may be cues to gender, while for women, the most important features may be linked to sexual activity, even when the actors are of the non-preferred gender or species.

In the present study, we wanted to address a new aspect of the relationship between gender and specific features displayed in sexual intercourse: the copulatory movement. To determine whether copulatory movement as such produces a sexual response, we examined the sexual response of androphilic women and gynephilic men to several videos depicting copulatory movement during sexual intercourse. We used two videos featuring humans and nine which featured non-human animals. By using videos of non-human penetrative copulation, we wanted to investigate the relative effect of copulatory movements on male and female sexual response separately from cues to (human) gender. Moreover, we want to examine a possible relationship between the genital and subjective sexual response and copulatory movements of non-human species differing in phylogenetic distance from humans. If androphilic women show a statistically significant increase in vaginal response to all non-human stimuli, regardless of their phylogenetic distance from humans, it would clearly indicate that copulatory movement is the most fundamental contextual feature eliciting a genital response. Based on previous findings, we hypothesized the following:

1. Unlike gynephilic men, androphilic women would demonstrate a statistically significant increase in genital response when exposed to non-human stimuli.
2. There would be no sex differences in subjective sexual response to any non-human stimuli.

## Method

### Participants

We conducted an a priori power analysis using GPower (Faul et al., 2009) for 2 × 11 mixed ANOVA with power (1* − β*) set at 0.95, *α* = 0.05, and smallest detectable effect size *f* = 0.15. With these parameters, the required number of participants is 52. Adding 10% for possible unexpected data loss and rounding up, we aimed at 60 split in two groups of heterosexual men and women.

Participants were recruited via the laboratory email list (https://www.sexlabnudz.cz/) and Facebook page (https://www.facebook.com/sexlabnudz) by posting an advertisement looking for “sexually active heterosexual men and women aged 18–45, who had not been diagnosed with any sexual or gynecological/urological issues and use no drugs that could affect their sexual functioning, for a study on psychophysiological responses to visual stimuli.”

We managed to recruit 69 participants, but data from five of them were lost due to a technical issue, and six persons willing to participate reported non-heterosexual orientation. The final sample size therefore consisted of 28 women (*M*_age_ = 26.00, SD_age_ = 6.78) and 30 men (*M*_age_ = 28.73, SD_age_ = 7.67), which is still well above the sample size required by power analysis.

### Measures

#### Sexual Orientation

Sexual orientation was assessed using the following wording: “I identify myself as…” Participants indicated their position on the 7-point Kinsey scale (from 0 “exclusively heterosexual” to 6 “exclusively homosexual”). Based on previous studies, we split the sample into separate groups of heterosexual and non-heterosexual men and women (Bártová et al., 2020; Chivers et al., 2007). Respondents on positions 0–2 were considered heterosexual (*N* = 58), while those on positions 3–6 were not included in the study (*N* = 6, one male and five females).

#### Penile Plethysmograph/Vaginal Photoplethysmography

Genital sexual arousal was assessed using a BIOPAC MP150 data acquisition unit and software AcqKnowledge, version 4.4.0. (BIOPAC Systems, Inc., Santa Barbara, CA). Men’s genital arousal was assessed via changes in air pressure using a volumetric penile plethysmography (PPG) pack consisting of an airtight glass tube and an air pressure meter. The signal was sampled at a rate of 2000 samples per second and low-pass filtered (300 Hz). The signal was recorded in centimeters of water (cmH2O). Women’s genital arousal was assessed via a change in vaginal pulse amplitude (VPA) using a VPG device consisting of a tampon-shaped acrylic vaginal probe. A silicone plate was attached to the cable of the VPG to ensure that the depth of the probe and orientation of the light source remained constant across all female participants. VPA signal was sampled at a rate of 2000 samples per second, band-pass filtered (0.5–30 Hz), and the amplitude of each pulse wave was recorded in millivolts (mV).

#### Self-Reported Sexual Arousal

Self-reported sexual arousal was assessed using the post-stimulus item “How sexually arousing did you find the video?” rated on a 9-point scale ranging from 1 (“Not at all”) to 9 (“Very”).

### Materials

#### Stimuli

A set of 11 video stimuli was selected for the study. Two video stimuli depicted human female-male and female-female penetrative sexual intercourse with prominent copulatory movements (e.g., scissoring), and nine video stimuli depicted non-human female-male penetrative sexual intercourse. Another video stimulus showed nature with trees and no animal or human activity. All videos were 60 s in length and soundless.

#### Selection Methods

Human stimuli were adjusted specifically for the purpose of this study. Two female and one male researcher previewed several commercially available adult films on Pornhub and EroticaX to compile a set of three candidate clips in each category: heterosexual stimuli and lesbian stimuli. Each video was then edited to a 60 s-length clip, which depicted consensual heterosexual/homosexual intercourse between one man and one woman or between two women in face-to-face sexual positions without any close-up of their genitals. These six clips were in a randomized order rated by 143 heterosexual men and women for a subjective rating of sexual arousal using a 10-point Likert-type scale ranging from 1 (“not at all”) to 10 (“very strongly”). We chose one heterosexual clip (*M* = 7.4, SD = 2.1) and one homosexual clip (*M* = 6.7, SD = 2.5) with the highest rating of sexual arousal in both heterosexual men and women.

Animal stimuli were likewise adjusted for the purpose of the study. Our goal was to use recent representations of species that have a common ancestor with humans, that is, mammals but also other species related through evolution, such as avians, reptiles, or insects (Zrzavý, 2006; for a visual scaling of phylogenetic relatedness, see https://vertlife.org/). Within each group, we looked for species whose copulatory movement is similar to humans (thrusting, speed, and rhythm) and easily perceptible. Three female and two male researchers previewed several videos from youtube.com and the film collection of the Faculty of Science to select two representatives for each group of species. Researchers independently assessed them for (1) the presence of copulatory movements (yes/no), and (2) the similarity of copulatory movements to human copulation. The final set of animal species included (ranked from the most to the least phylogenetically related to humans, Zrzavý, 2006): chimpanzees (*Pan troglodytes*), western lowland gorillas (*Gorilla gorilla gorilla*), guinea pigs (*Cavia porcellus*), European hares (*Lepus europaeus*), lions (*Panthera leo*), mountain zebras (*Equus zebra*), budgerigars (*Melopsittacus undulatus*), common five-lined skinks (*Plestiodon fascinatus*), and bush crickets (*Metaplastes ornatus*). Each video was edited to a 60 s length clip without sound, which depicted the copulation of the animal pair in rear-entry or side-by-side sexual positions without a close-up of their genitals. Where animal copulation lasted less than 60 s, copulatory movement of the same animal pair was looped for a sufficient length of time.

A neutral stimulus depicted nature with trees and bushes and close-ups of leaves with no animal or human activity. The video was taken from an online Czech documentary (provided by Czech Television).

#### Distractors

A set of 10 cartoon seek-and-find pictures was used as distractors. All pictures were downloaded from the artist Dudolf (Dudás, 2021). These pictures include a large number of elements, such as football balls or cartoonish bats. Viewers are expected to explore the picture and find an element that is broadly visually similar but depicts something clearly different, e.g., a panda head or a black cat. These pictures were selected as distractors because, while not cognitively challenging, their solution within the relatively short time set in the study required considerable effort and attention.

### Procedure

Prior to laboratory testing, all interested participants were screened for inclusion criteria (i.e., being aged 18–45 years and sexually active without any sexual health problems) and filled a battery of online questionnaires. Upon arrival, the experimenter explained the study procedure and obtained informed consent. Next, female participants were instructed in the proper use of the VPG. When the experimenter left the room, each female participant inserted the vaginal probe on her own. Participants were seated in a comfortable chair, about 1 m from a screen. Each male participant set the PPG device with the researcher’s assistance. A researcher of their own sex (men by a male researcher, women by a female researcher) assisted all participants. Once set with a device, each participant was instructed to attend to the screen, and the stimulus presentation began. The VPA/PPG recording started with a 3-min baseline measurement, during which participants were presented with natural scenes. Subsequently, all other 11 videos were presented in a randomized order. After each stimulus, participants assessed their subjective sexual arousal and completed the distraction tasks. Each distractor task lasted 30 s, and their order was also randomized. After the measurements, participants were debriefed and given a small set of sex toys, condoms, and lubricants. The whole procedure took about 30 min. The study was approved by the Research Ethics Committee of the Faculty of Humanities, Charles University.

### Data Analysis

Following standardized procedures, any movement artifacts (defined by sudden changes in pulse amplitude) were visually identified and manually removed (Prause & Janssen, 2005). The mean peak-to-peak amplitude (VPG) and mean values (PPG) were calculated from the whole 60 s presentation of each stimulus. Data management and statistics were done in R 4.0.5 (R Core Team, 2021), *cocor* package 1.1.3 (Diedenhofen & Much, 2015), and JASP 0.16.1 (JASP Team, 2022).

Because PPG and VPG give results in different units, the data were standardized within subjects using z-scores to control for interindividual differences and to enable a direct comparison of the relative magnitude of genital response to a particular stimulus (Harris et al., 1992). Given the nature of the standardization, the main effect of sex differences will always be 0, and as such, it is uninterpretable and reported only for the sake of completeness.

A two-way mixed ANOVA with one within-subject factor (category: nature, budgerigars, crickets, gorillas, guinea pigs, hares, heterosexual human pair, chimpanzees, lesbian human pair, lions, skinks, zebras) and one between-subject factor (sex: male, female) was performed for the standardized genital response and for subjective arousal. The relation between subjective and genital arousal was tested using the Pearson correlation coefficient computed across all participants and conditions. For all tests, the alpha level of statistical significance was set to 0.05, and effect sizes were calculated.

## Results

### Genital Response

Mauchly’s test did indicate a violation of the sphericity assumption: *χ*^2^(65) = 123.97, *p* < .001, which is why the degrees of freedom were corrected using Greenhouse–Geisser estimates of sphericity (*ε* = 0.67).

There was a significant main effect of category: *F*(7.38, 413.27) = 35.63, *p* < .001, *ω*^2^ = 0.38, *µ*^2^*p* = 0.39. Post-hoc comparisons with Holm correction revealed that genital responses to heterosexual human pair (*M* = 1.47, SE = 0.11, SD = 0.83, 95% CI [1.26, 1.69]) and lesbian human pair (*M* = 1.12, SE = 0.13, SD = 0.96, 95% CI [0.87, 1.36]) were significantly greater than genital responses to other video stimuli (*M* = from − 0.42 to 0.01), all *p* < .001, but not significantly different from each other (*p* = .724). Genital responses did not differ among other stimuli (*p* > .05). The main effect of sex was nonsignificant, *F*(1, 56) = 0, *p* = 1, *ω*2 = 0, *µ*2*p* = 0.

**Fig. 1:**
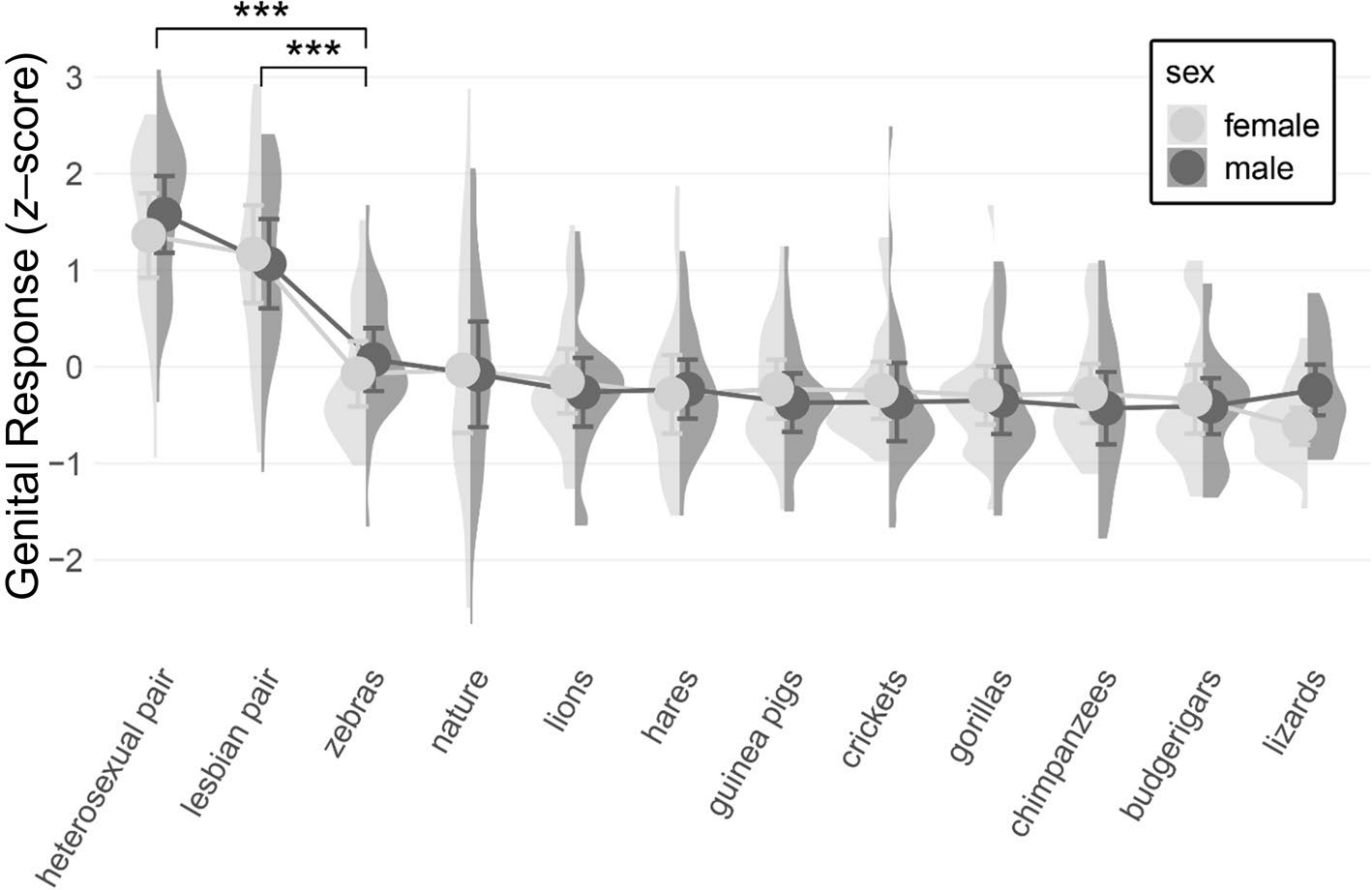
Male and female standardized genital response, means with SD, density plots

The interaction effect between category and sex was nonsignificant, *F*(7.38, 413.27) = 0.63, *p* = .74, *ω*2 = 0, *µ*2*p* = 0.01. This shows that there was no significant difference in men’s and women’s relative genital reactions and that their genital reactions did not differ based on the stimulus category. For comparisons, see Fig. 1 and Table 1.

**Table 1:** Male and female genital arousal for all stimuli with means (SD) in *z*-scores

Stimulus	Males	Females
Heterosexual pair	1.58 (0.80)	1.36 (0.88)
Lesbian pair	1.07 (0.92)	1.17 (1.01)
Lions	− 0.26 (0.71)	− 0.15 (0.67)
Gorillas	− 0.35 (0.70)	− 0.29 (0.61)
Zebras	0.08 (0.65)	− 0.07 (0.68)
Budgerigars	− 0.41 (0.58)	− 0.34 (0.71)
Lizards	− 0.24 (0.53)	− 0.61 (0.38)
Chimpanzees	− 0.43 (0.75)	− 0.28 (0.61)
Hares	− 0.23 (0.61)	− 0.28 (0.82)
Crickets	− 0.36 (0.81)	− 0.24 (0.59)
Guinea pigs	− 0.37 (0.61)	− 0.23 (0.61)
Nature	− 0.08 (1.09)	− 0.03 (1.30)

### Subjective Response

Mauchly’s test indicated a violation of the sphericity assumption; *χ*^2^(54) = 346.01, *p* < .001. Since sphericity was violated (*ε* = 0.37), we report Greenhouse–Geisser corrected results.

There was a significant main effect of category: *F*(3.92, 219.46) = 214.89, *p* < .001, *ω*2 = 0.74, *µ*2*p* = 0.79. Post-hoc comparisons with Holm correction showed that the largest subjective arousal was elicited by depictions of heterosexual (*M* = 6.72, SE = 0.26, SD = 2.00, 95% CI [6.21, 7.24]) and lesbian human pairs (*M* = 5.79, SE = 0.30, SD = 2.25, 95% CI [5.22, 6.37]). The level of arousal differed both between these two human stimuli (*p* < .001) and between these two stimuli and the rest of the videos (*p* < .001). From the other videos, the largest subjective arousal was elicited by lions (*M* = 2.07, SE = 0.16, SD = 1.20, 95% CI [1.76, 2.38]), followed by gorillas (*M* = 1.78, SE = 0.15, SD = 1.17, 95% CI [1.47, 2.08]), and zebras (*M* = 1.74, SE = 0.10, SD = 0.74, 95% CI [1.55, 1.93]). The smallest subjective arousal was elicited by budgerigars (*M* = 1.48, SE = 0.11, SD = 0.86, 95% CI [1.26, 1.70]), lizards (*M* = 1.43, SE = 0.10, SD = 0.75, 95% CI [1.24, 1.62]), chimpanzees (*M* = 1.40, SE = 0.08, SD = 0.62, 95% CI [1.24, 1.56]), hares (*M* = 1.35, SE = 0.08, SD = 0.58, 95% CI [1.20, 1.49]), bush crickets (*M* = 1.29, SE = 0.07, SD = 0.56, 95% CI [1.15, 1.44]), nature (*M* = 1.21, SE = 0.07, SD = 0.55, 95% CI [1.06, 1.35]), and guinea pigs (*M* = 1.12, SE = 0.04, SD = 0.33, 95% CI [1.04, 1.21]).

**Fig. 2:**
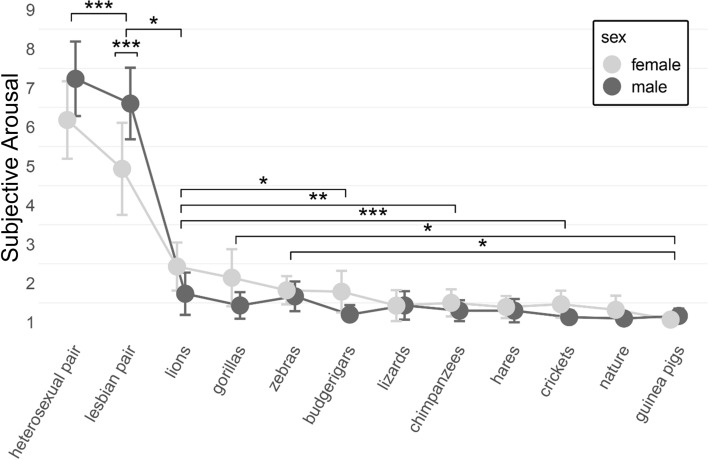
Male and female subjective arousal, means with SD

Lions were rated as significantly more arousing than budgerigars (*p* = .05), lizards (*p* = .01), chimpanzees (*p* = .01), hares (*p* < .01), or crickets, nature, and guinea pigs (all *p* < .001), but not as significantly more arousing than gorillas or zebras (both *p* = 1). Both gorillas and zebras were significantly more arousing than only the guinea pigs (*p* = .01 and *p* = .02, respectively) but not significantly more arousing than the rest of the stimuli (*p* > .05). There was no difference between the evaluations of gorillas and zebras (*p* = 1). Budgerigars, lizards, chimpanzees, hares, crickets, nature, and guinea pigs likewise did not elicit significantly different levels of subjective arousal (*p* > .05). For comparisons, see Fig. 2 and Table 2. The main effect of sex was nonsignificant: *F*(1, 56) = 0.01, *p* = .92, *ω*2 = 0, *µ*2*p* = 0.

**Table 2:** Male and female subjective arousal for all stimuli with means (SD)

Stimulus	Males	Females
Heterosexual pair	7.23 (1.91)	6.18 (1.98)
Lesbian pair	6.60 (1.83)	4.93 (2.36)
Lions	1.73 (1.08)	2.43 (1.23)
Gorillas	1.43 (0.68)	2.14 (1.46)
Zebras	1.67 (0.76)	1.82 (0.72)
Budgerigars	1.20 (0.48)	1.79 (1.07)
Lizards	1.43 (0.73)	1.43 (0.79)
Chimpanzees	1.30 (0.54)	1.50 (0.69)
Hares	1.30 (0.60)	1.39 (0.57)
Crickets	1.13 (0.35)	1.46 (0.69)
Guinea pigs	1.17 (0.38)	1.07 (0.26)
Nature	1.10 (0.31)	1.32 (0.72)

Subjective arousal was rated on a 9-point scale ranging from 1 (“Not at all”) to 9 (“Very”)

There was a significant interaction effect between category and sex: *F*(3.92, 219.46) = 7.59, *p* < .001, *ω*2 = 0.08, *µ*2*p* = 0.12. Post-hoc comparisons with Holm correction revealed that men (*M* = 6.60, SE = 0.33, SD = 1.83, 95% CI [5.94, 7.26]) reported larger subjective arousal to lesbian stimulus than women did (*M* = 4.93, SE = 0.45, SD = 2.36, 95% CI [4.06, 5.80]), *p* < .001, *d* = 1.55. Subjective arousal in response to a heterosexual stimulus was also significantly larger in men (*M* = 7.23, SE = 0.35, SD = 1.91, 95% CI [6.55, 7.92]) than in women (*M* = 6.18, SE = 0.38, SD = 1.98, 95% CI [5.44, 6.91]), *p* < .001, *d* = 0.98. There was no significant difference between men and women in the level of their subjective arousal in response to any of the other stimuli. For comparisons, see Fig. 2.

### Genital and Subjective Correlation

**Fig. 3:**
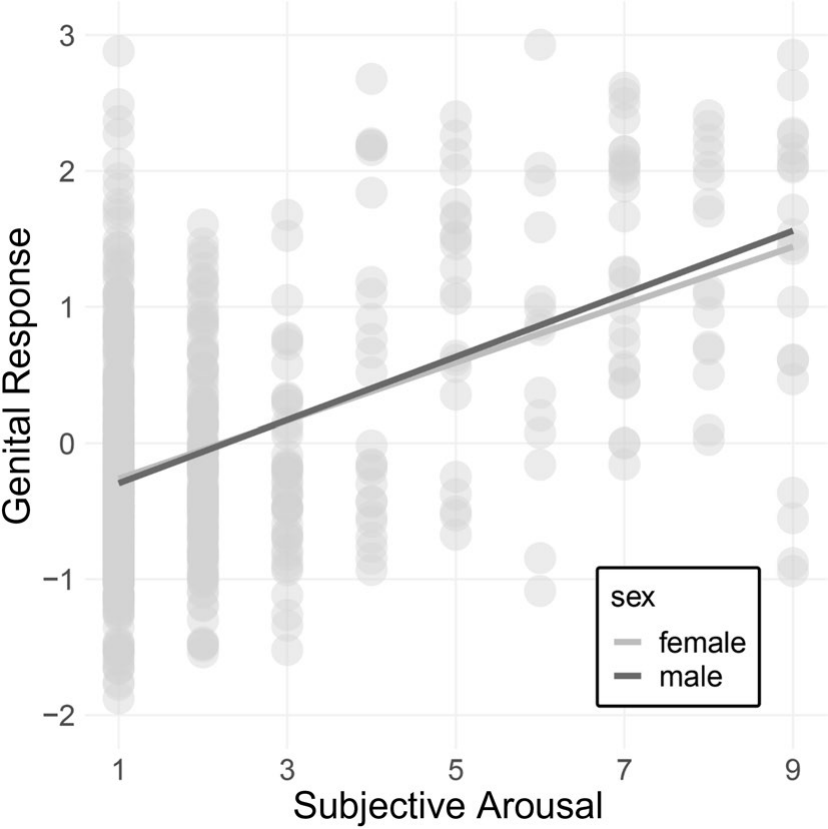
Regression lines for subjective and genital arousal in males and females

There was a statistically significant correlation between genital and subjective arousal in both sexes: *r*(694) = .51, *p* < .001, 95% CI [0.45, 0.56]. When analyzed separately, there was a statistically significant correlation between genital and subjective arousal in both men (*r*(358) = .57, *p* < .001, 95% CI [0.49, 0.64]) and women (*r*(334) = .43, *p* < .001, 95% CI [0.34, 0.52]). The correlations also significantly differed from each other, *Z* = 2.40, *p* = .02. For correlations, see Fig. 3.

## Discussion

This is the first study to examine the specific contribution of visually presented copulatory movements to genital and subjective arousal in androphilic women and gynephilic men. Contrary to our initial hypothesis, an increase in genital arousal was observed only when androphilic women were presented with stimuli depicting human female-male and female-female penetrative sexual intercourse. In other words, women showed a specific pattern of genital arousal in response to both human and non-human stimuli. As hypothesized, gynephilic men showed neither genital nor subjective arousal in response to non-human sexual stimuli. The level of genital arousal response was similar across all non-human stimuli in both women and men. Similarly, self-reported sexual arousal was highest for the female-male and female-female sexual stimuli for both women and men, although men reported larger subjective arousal in response to the heterosexual and lesbian stimuli than women did. These findings are consistent with previous studies. Also, we found no significant difference in the level of subjective arousal in response to any of the non-human stimuli in either men or women. Genital responses and self-reported sexual arousal were positively correlated in both women and men.

These findings are not consistent with the hypothesis that copulatory movements in themselves (in the absence of other contextual cues, such as gender, sexual vocalization, etc.) are the most fundamental feature that elicits a non-specific genital response in androphilic women.

In contrast to the Chivers and Bailey (2005) and Chivers et al. (2007) studies, we found no increase in the genital arousal of androphilic women in response to female and male chimpanzees or other non-human species engaging in sexual activity. This might be due to the different attributes of sexual activity displayed in human as opposed to non-human sexual stimuli.

The stimuli used in the present study were not identical to those used by Chivers and Bailey (2005) or Chivers et al. (2007). Among other things, we used visual sexual stimuli of human and non-human penetrative copulation without sound, while the stimuli used by Chivers and Bailey (2005) and Chivers et al. (2007) were depictions of human and bonobo penetrative copulation, which did include sexual vocalizations. Although the exclusion of sexual vocalizations is a strength of the current study (because it eliminated a particular contextual cue), it is possible that these features, i.e., the vocalization in non-human sexual stimuli in Chivers and Bailey (2005) and Chivers et al. (2007), were responsible for the non-specific genital response to bonobo copulation stimuli observed in previous studies. Research that has used audio-visual sexual stimuli, which include sexual vocalizations, suggests that the auditory aspect augments both subjective and genital responses in men (Gaither & Plaud, 1997) but not in women (Polan et al., 2003). Other data suggest that the inclusion of vocalizations amplifies self-reported sexual arousal in both women and men (Pfaus et al., 2006). We, however, believe that audio-visual sexual stimuli occupy a greater number of sensory channels and thereby attract greater attention to sexual stimuli—which in turn leads to a greater sexual response.

Previous studies have also shown that women tend to identify with actors in sexual stimuli, i.e., that they imagine themselves in the depicted sexual interactions (Chivers, 2017; Symons, 1979). It seems that women who watch videos showing other people enjoying sexual pleasure can identify with these cues and project themselves into such scenarios (Chivers, 2017). However, the study by Bossio et al. (2014) showed that both men and women imagine themselves as participants in their preferred stimuli rather than the observer, although androphilic women rate higher observer stance scores for their non-preferred stimuli over preferred stimuli. Moreover, women identifying with and passively observing the actors (taking an observer and participant stance) in erotic stimuli predicts women’s subjective but not genital arousal. By comparing the human to non-human stimuli, we can speculate that images of various non-human animal species engaging in copulation perhaps do not lead to women’s as well as men’s identification with cues to sexual pleasure. In light of these considerations, one could speculate that category-nonspecific genital response in androphilic women might emerge when other contextual cues (such as sexual vocalizations, sexually aroused genitals, or faces) are present.

Cognitive models of sexual response propose that a positive affect directs attention to sexual stimuli, thereby increasing sexual response, while a negative affect interferes in the processing of sexual cues, resulting in a lower sexual response (see Barlow, 1986). It seems likely that women do not process non-human sexual stimuli as ‘sexual’, which results in a smaller positive affect during the stimulus presentation and ultimately in a smaller sexual response. This hypothesis would also explain the concordance between the levels of genital and subjective arousal in androphilic women in our study. This higher concordance among women may reflect their experience of a negative affect while watching non-human copulation that is perceived as nonsexual. This is consistent with the information-processing model of sexual arousal (Janssen et al., 2000), which incorporates two pathways: an unconscious pathway, responsible for generating a genital response to sexual stimuli, whereby the integration of sexual meanings in implicit memory and motor response triggers an automatic genital response, and a conscious pathway, responsible for subjective sexual arousal, whereby the presence or absence of genital response and assessment of sexual stimuli (e.g., as sexual or nonsexual, positive or negative) orients attention to the stimulus, which then leads to subjective sexual arousal. If men and women derive part of their subjective sexual arousal from their genital response (in a feedback loop), it seems possible that the men and women in the current study were producing significantly higher genital responses to human sexual stimuli because they found them subjectively sexually arousing, and vice versa. In a similar way, previous studies found that men and women report lower sexual arousal (both genital and self-reported) to sexual stimuli when they experience a higher level of aversion or disgust (Andrews et al., 2015; Borg & de Jong, 2012; Koukounas & McCabe, 1997). In line with these findings, the sexual stimuli we have used, especially those displaying non-human copulation, might have elicited feelings of aversion or disgust and thus become less capable of eliciting sexual arousal.

The non-specific genital response to female-male bonobo copulation stimuli found in previous studies by Chivers (2005, 2007) was explained as a reflexive vaginal response to any sexual stimuli. This explanation is in line with Laan and Everaerd’s (1995) idea that women have evolved a rapid reflex-like genital response to sexual stimuli. A reflexive genital vasocongestion in response to any cues to sexual activity may serve as a protective mechanism that protects the female genital organs from potential injury (it is known as the preparation hypothesis; see Suschinsky & Lalumière, 2011a). The preparation hypothesis also suggests that only salient sexual cues elicit a genital response. If non-human sexual stimuli were in our study perceived as nonsexual, it is not surprising that both androphilic women and gynephilic men did not sexually respond. Moreover, the preparation hypothesis offers an explanation of women’s genital response to sexual stimuli based only on laboratory-based experiments (see Lalumière et al., 2022 for a review). From an evolutionary perspective, it is unlikely for women to have sexual intercourse with males of a different species. It is thus not adaptive to develop an automatic protective genital response to depictions of non-human sexual acts given that human–animal sexual behavior is not reproductively motivated.

### Limitations and Future Directions

This study is the first to examine directly the effect of copulatory movements on men’s and women’s sexual responses. Few studies so far examined the specific features of sexual stimuli that evoke genital and self-reported sexual arousal (e.g., Spape et al., 2014). The strength of our study is that we used sexual stimuli with limited contextual cues (such as sexual vocalization or display of sexually aroused genitals). This restriction of sexual cues can provide greater insight into the role of copulatory movements as a trigger of sexual response.

One of the limitations of our study is that homosexual stimulus displaying male-to-male sex is missing. Based on the previous studies, we aimed to include the human videos that were likely to elicit the strongest sexual response in both androphilic women and gynephilic men, that is, heterosexual and lesbian human sex (e.g., Chivers & Bailey, 2005). But having data on male-male sex would yield a better picture of gender-non/specific sexual responses in men and women.

Another potential methodological limitation of our study concerns the animal stimuli we used. As we considered the phylogenetic distance to humans as a relevant feature of genital and subjective arousal, it would be worth asking participants to rank the non-human species in terms of perceived proximity to the human species. A person’s perceptions of relative closeness in the phylogenetic web might affect an individual’s arousal when watching copulation.

Gender differences reported in the study may be limited to those individuals who volunteer for sexual psychological research. A recent study suggested that such volunteers tend to have a greater sexual experience and more positive sexual attitudes than people who would not volunteer for such studies (Dawson et al., 2019). Nevertheless, it has also been reported that differences between volunteers and non-volunteers do not influence the gender-specific features of sexual arousal patterns (see Chivers et al., 2004).

It must also be noted that our study was limited to heterosexual individuals: future studies should examine the effect of copulatory movements on the sexual responses of homosexual men and women.

### Conclusion

This is the first study to examine the cue-specific contribution of copulatory movements on the genital and subjective sexual response of androphilic women and gynephilic men. Our findings suggest that copulatory movements displayed in non-human sexual stimuli do not constitute a contextual cue sufficient to elicit a genital response. Previous research has shown that patterns of women’s sexual arousal response are sensitive to stimulus cues and context. If contextual cues are limited, as they were in this study, women might demonstrate a specific sexual response pattern. Based on a comparison of human to non-human stimuli, we can speculate whether viewing of sexual activity (and copulatory movement) in a different animal species is a sufficient trigger of sexual response. More research is required to further assess the role of copulatory movements as a potential sexual trigger of a cue-specific response in androphilic women. These results continue to add to the rich body of literature on the cue-specificity of sexual arousal in women and men, whereby this research is of practical importance for our understanding of the stimulus features associated with sexual response.
